# Methods of MicroRNA Promoter Prediction and Transcription Factor Mediated Regulatory Network

**DOI:** 10.1155/2017/7049406

**Published:** 2017-06-05

**Authors:** Yuming Zhao, Fang Wang, Su Chen, Jun Wan, Guohua Wang

**Affiliations:** ^1^State Key Laboratory of Tree Genetics and Breeding, Northeast Forestry University, Harbin, Heilongjiang, China; ^2^Information and Computer Engineering College, Northeast Forestry University, Harbin, Heilongjiang, China; ^3^School of Computer Science and Technology, Harbin Institute of Technology, Harbin, Heilongjiang, China; ^4^Department of Medical and Molecular Genetics, Indiana University School of Medicine, Indianapolis, IN, USA

## Abstract

MicroRNAs (miRNAs) are short (~22 nucleotides) noncoding RNAs and disseminated throughout the genome, either in the intergenic regions or in the intronic sequences of protein-coding genes. MiRNAs have been proved to play important roles in regulating gene expression. Hence, understanding the transcriptional mechanism of miRNA genes is a very critical step to uncover the whole regulatory network. A number of miRNA promoter prediction models have been proposed in the past decade. This review summarized several most popular miRNA promoter prediction models which used genome sequence features, or other features, for example, histone markers, RNA Pol II binding sites, and nucleosome-free regions, achieved by high-throughput sequencing data. Some databases were described as resources for miRNA promoter information. We then performed comprehensive discussion on prediction and identification of transcription factor mediated microRNA regulatory networks.

## 1. Introduction

MicroRNAs (miRNAs) are small noncoding RNAs with about 22 nucleotides, which are transcribed by noncoding DNA sequences [1, 2]. MiRNAs are disseminated throughout the genome. They were found either in the intergenic regions or in the intronic sequences of protein-coding genes. It has been known that miRNAs are key elements in many species, such as human and mouse, to function in posttranscriptional gene regulation. One single miRNA can influence one-third of human genome by potentially regulating thousands of genes at the same time [3]. Similar to protein-coding genes, miRNAs were also regulated by transcription factors (TFs) at transcription level. Uncovering the transcriptional mechanisms of miRNAs themselves can help people better understand regulatory networks of gene expression.

The promoters of genes are important regions, bound by different regulatory elements to start and regulate the transcription [4, 5]. Locating the promoter regions of genes is crucial for revealing the transcriptional mechanism. It remains difficult to define miRNA promoters and understand how TFs regulate downstream miRNAs. The classical features of promoter regions, including signal, context, and structure features, can be used to recognize miRNAs from other sequences [6]. However, only a fraction of the human miRNAs have their transcription start sites (TSSs) confirmed. Insufficient knowledge of the TSSs of miRNA genes limited our ability to study the transcriptional mechanism and the regulatory function of miRNAs. While most of promoter prediction methods based on the promoters of protein-coding genes may not be suitable for miRNA genes, it is required to develop promoter prediction methods special for miRNA genes.

In recent years, more and more prediction models have been developed to identify the miRNA promoters [7–12]. These studies utilized genome sequence features or took advantage of the high-throughput sequencing technology to identify the putative promoter regions of miRNA genes. In this article, we reviewed algorithms of miRNA promoter recognition based on genome sequence features, histone markers, RNA Pol II binding, and nucleosome-free regions achieved from high-throughput sequencing data, respectively. We performed a comparative analysis on these models and corresponding identified miRNA promoter regions. In order to better understand the regulatory mechanisms of miRNA, more and more databases have been developed to collect miRNA promoter regions by integrating different prediction models. We also evaluated several databases collecting such miRNA promoter information. In the last part, we discussed the TF-miRNA regulatory networks either predicted by computational methods or derived by high-throughput experiments.

## 2. A Survey on Methods for MiRNA Promoter Regions Prediction

The prediction of miRNA promoters is significant for constructing the regulatory network of TF-miRNA or miRNA gene and further understanding the regulatory function of miRNAs. Several most popular prediction approaches used traditional genome sequence features, either individual one or mixed features, whereas more and more methods adopted next-generation sequencing (NGS) data to employ the information of histone markers, RNA Pol II binding sites, and nucleosome-free regions. Below is a survey on some representative methods.

### 2.1. Prediction Methods Using Traditional Genome Sequence Features

#### 2.1.1. Individual Genome Sequence Features-Based Method

At the early beginning, researchers used one single genome sequence feature, expressed sequence tags (ESTs), to predict miRNA promoter regions. ESTs technology directly originated from the human genome project to construct the genetic map of genome. Many intergenic miRNAs are transcribed as pri-miRNAs. Gu et al. successfully predicted the location of pre-miRNAs by mapping the ESTs to the long flanking sequences. They then used EST-extension method to predict the location of about tens of pri-miRNA [13]. By comparing promoters of known miRNAs and protein-coding genes, Zhou et al. discovered that the transcriptional mechanism of miRNAs was similar to that of protein-coding genes in that both miRNAs and protein-coding genes were transcribed by RNA Pol II [14]. By relying on the sequence feature of known Pol II promoters, they extracted all possible k-mers as such features and used WordSpy algorithm [21, 22] to discover sequence motifs. Then they developed a new approach, CoVote [14], to predict unknown core promoters of miRNAs. CoVote was based on the decision tree algorithm followed by training well-known Pol II promoters compared to randomly selected sequences. The method has been proved to create good predictions by being applied on four species,* C. elegans*,* H. sapiens*,* A. thaliana*, and* O. sativa*.

#### 2.1.2. Mixed Genome Sequence Features-Based Method

As we discussed previously, early modeling of miRNA promoters focused only on individual sequence features [13, 14]. While genome sequences have plenty of different features, combining these features can improve the accuracy of miRNA promoters' prediction. Genome sequences are composed of four bases, A, C, G, and T. The different assemblies of four bases form the sequence features of genome, such as TATA box, CAAT box, and GC box. Using the TRANSFAC weight matrices of TATA box, CAAT box, and GC box, Fujita and Iba utilized an entropy-based calculation to search the promoter of miRNA genes, which was implemented in the aligned and conserved blocks that contained miRNA hairpin regions [15]. To verify this method, they predicted 59 core promoter regions for 79 miRNAs, which were conserved between human and chicken or between human and zebrafish.

Furthermore, by incorporating several different sequence features, Bhattacharyya et al. used SVM model to predict TSSs of intergenic miRNA [17]. They extracted a large number of sequencing features in their study, such as N-mer features, palindromic features, special features, and CpG island based features. Those miRNA TSSs experimentally verified in previous studies were used to design the SVM classification model. Then they used well-trained complex AMOSA-SVM model to recognize unknown miRNA TSSs.

Similar with the above approach, Marsico et al. proposed a new approach, named PROmiRNA [16], based on a semisupervised statistical model. First, the TSS clusters of pre-miRNAs were generated. Second, they normalized the TSS clusters by removing the TSS clusters overlapping with the start of other protein-coding transcripts or spanning exon regions. Third, the sequence features, including CpG density, conservation score, TATA box affinity, and normalized tag counts, were calculated around the putative TSSs regions and the random regions. The region with a higher probability of being a promoter region than being a nonpromoter region is determined as a potential promoter region.

The summary of the prediction results of these methods is shown in Table 1, including the number of the putative miRNA promoter region of every method using genome sequence features. However, these methods using genome sequence features still have limitations in different tissues and species and hence their accuracy is not high enough.

### 2.2. Prediction Methods Using High-Throughput Sequencing Data

With rapid development of the NGS technology, whole genome and exome sequencing provides researchers with the opportunity to deal with the complex transcriptional and regulatory problem. Many sequencing technology such as RNA-seq and ChIP-seq can obtain the detailed information of genes, TFs, histone markers, nucleosome-free regions, and so on. Nowadays, more and more high-throughput sequencing data about miRNA expression have been collected, providing the opportunity to more accurately identify the TSS of miRNA and predict miRNA promoters.

#### 2.2.1. Histone Markers-Based Method

Histone modifications represent different chromatin states. The NGS technology, ChIP-seq, is widely used to recognize locations of histone modifications. Many previous studies have showed that H3K4me3 was enriched in miRNA promoter regions, similar to that in the promoters of protein-coding genes. Therefore, using the data of histone modifications becomes popular to predict miRNA promoters. Marson et al. used the ChIP-seq data of H3K4me3 containing genomic enriched loci of H3K4me3, to predict the TSSs of miRNA genes in human and mouse genomes [9]. As a consequence, almost 80% of miRNAs promoters were identified in human and mouse genome.

In 2009, Wang et al. developed a computational program, called CoreBoost_HM, which combines several DNA features with histone modification [8]. The DNA features included the core promoter elements score, density of transcription factor binding sites (TF BSs), Markovian log-likelihood ratio scores, and N-mer frequencies. The boosting algorithm was used to model these feature data to predict the core promoters of miRNA genes. This combination of DNA features and histone modification improved the accuracy of prediction of miRNA promoters.

Different types of histone markers exhibit different patterns and functions in the genome sequences. In a previous study, we used nine different histone markers to predict miRNA promoters in* Arabidopsis* [18]. These histone markers included H3K4me2, H3K4me3, H3K9Ac, H3K9me2, H3K18Ac, H3K27me1, H3K27me3, H3K36me2, and H3K36me3. The RPM (reads per million per 100 bp bin) values of these nine histone modifications were extracted from corresponding ChIP-seq experiments for each known and unknown promoter region, indicating their binding patterns on these regions, respectively. The SVM model was trained based on these datasets, by using radial basis function (RBF) as the kernel function. Finally we identified TSSs of most miRNA genes and analyzed distinct histone patterns around the predicted TSSs of miRNA genes.

#### 2.2.2. Pol II Binding-Based Method

It is believed that most miRNA genes are also transcribed by RNA polymerase II (Pol II), just like the protein-coding genes [23, 24], although some exceptions exist [25]. The binding of Pol II on the genome sequences can be used to investigate the transcriptional mechanism of miRNA genes. In order to start the transcription, Pol II always binds in close proximity to the TSSs of genes. In other words, Pol II binding pattern may be a key element of the promoter prediction. To better make out the transcriptional mechanism of miRNAs, Corcoran et al. performed ChIP-chip experiment for Pol II [11]. Based on SVM, they developed an efficient method for predicting core promoter, called CPPP. They successfully applied these tools to predict miRNA TSSs and analyzed the transcriptional mechanisms of miRNA genes.

Using genome-wide Pol II binding patterns, Wang et al. designed a computational approach to identify the promoter regions of miRNA genes [10]. A statistical model was developed to simulate the binding patterns of Pol II around the known TSSs of highly expressed protein-coding genes. Utilizing maximum likelihood estimation, they selected the best parameters that described the binding patterns of Pol II around TSSs. According to the assumption that the Pol II distribution around the TSSs of miRNA genes is similar to that around the TSSs of protein-coding genes, the upstream regions of miRNAs were then scanned to search for the regions with similar simulated Pol II binding patterns. These regions were inferred as the putative TSSs of miRNA genes.

To predict the promoter of* Arabidopsis* miRNAs, Zhao et al. performed ChIP analysis of Pol II in* Arabidopsis* using a genome tiling microarray based on the function of Pol II [19]. Using the approach of sliding window, the Pol II binding profiles around the known TSS of 59 miRNA genes were obtained. To predict TSSs for miRNA genes, they developed a procedure with three major steps: (i) setting the loci in the upstream of the Pol II signal intensity valley as an initial start position; (ii) using motif matcher to search for TATA box around the start point; (iii) scanning the same region by using the transcription initiation motifs verified by experiments. Then different TSS was identified for each miRNA gene based on the different position of TATA box.

The previous studies have indicated that H3K4me3, Pol II, and TFs played important roles in regulating the expression of miRNA genes. While most of the above studies used just one type of feature to identify miRNA TSSs, Georgakilas et al. incorporated three different types of features, including H3K4me3 peaks, Pol II peaks, and DNaseI peaks data, to construct a method, named MicroTSS, for predicting miRNA TSSs [20]. They first utilized these three features to train three SVM models using libsvm v3.0. Then MicroTSS was developed by combining H3K4me3, Pol II, and DNaseI occupancy models together. Considering that miRNA genes had the similar expression mechanism with protein-coding genes, they applied MicroTSS acquired by protein-coding genes data to predict miRNA TSSs.

#### 2.2.3. Nucleosome-Free Region-Based Method

It is known that TFs generally bind in nucleosome-free regions, which is proximity to the TSSs, to activate downstream genes. Promoter regions usually reveal some significant features, such as high evolutionary conservation, nucleosome-depleted regions, CpG islands, TFBS motif within regions, and specific histone modification containing H3K4me3, H3K9ac, and H3K14ac. Based on the assumption that a nucleosome-free region within the ChIP-enriched site may contain a TSS, Ozsolak et al. utilized these characteristic features to develop a scoring function to predict miRNA promoters [7]. The center of the valley with the highest score was defined as the putative TSS.

**Figure 1:**
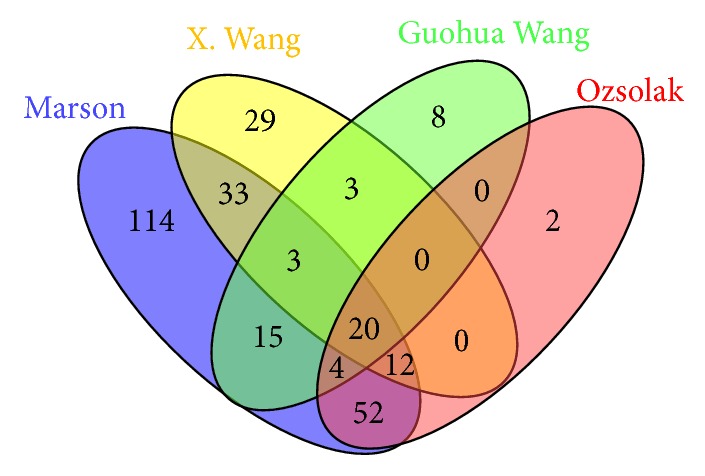
*Vann diagram of putative promoter regions*. The promoter was predicted by four models proposed by Marson et al., X. Wang et al., Ozsolak et al., and Guohua Wang et al., respectively.

We make a comparison of four models using high-throughput sequencing data [7–10].  Figure 1 shows the Venn diagram of the prediction promoter regions of these four models. The putative miRNA promoter regions of the model used by Marson et al. are chosen as the criterion to compare with other three putative results. Since genomic coordinates of datasets of Marson et al. are based on GRCh37/hg19, the other three datasets are based on NCBI36/hg18, the liftOver program obtained from the UCSC Genome browser [26] was applied to convert genomic loci of datasets of Marson et al. into NCBI36/hg18. There are 20 common putative miRNA promoter regions predicted by these four models, shown in Table 2.

## 3. The Database of miRNA Promoter Construction

There were not many studies on miRNA promoter at the early stage. Moreover, most reports focused on only a few miRNAs in special species or tissues. In recent years, more and more investigations about the prediction of miRNA promoter regions have appeared. In order to make researchers have a comprehensive understanding of miRNA genes expression and functions, there are increasing numbers of databases that collect the promoter information of different miRNA and provide analysis tools to researchers.

Bhattacharyya et al. constructed a database, named miRT, which accumulated the validated miRNA TSSs of the previous studies [27]. They searched PubMed extensively to obtain the information about miRNA TSSs. The miRT database covers 670 TSS loci of 588 miRNAs with a minimum support value of one, which includes 206 inter-miRNAs and 382 intra-miRNAs. Some miRNAs may have multiple TSSs. The miRT database is available at http://www.isical.ac.in/~bioinfo_miu/miRT/miRT.php.

Chien et al. constructed the database, miRStart, a novel resource of human miRNA TSSs [12]. It systematically incorporates three significant datasets, including CAGE tags, TSSs seq data, and H3K4me3 ChIP-seq data, derived from TSS-relevant experiments to identify TSSs of miRNAs. In general, a high-confidence TSS is recommended for each miRNA genes based on a SVM training model. Through the database, users can define their preferable miRNA TSSs according to the straightforward display of experimental TSS signals. In total, miRStart involves 940 human miRNAs. Among them, 352 miRNAs are inter-miRNAs, and 588 miRNAs are intra-miRNAs. The miRStart database is freely available at http://mirstart.mbc.nctu.edu.tw/.

Panagiotis Alexiou et al. constructed miRGen database, providing the promoter positions of miRNA genes in human and mouse, and their regulation by TFs [28]. The data are supported by experimental results. The information about microRNA coding transcripts, such as promoter regions, is supported by four literature sources: (i) Corcoran et al. [11], (ii) Landgraf et al. [29], (iii) Ozsolak et al. [7], and (iv) Marson et al. [9]. In total, there are 812 human miRNAs and 386 mouse miRNA coding transcripts' information stored in this database. Among these, 423 miRNAs are intra-miRNAs. In addition, this database shows binding sites of some TFs on the promoter regions of miRNAs and the information about SNPs. The miRGen database is freely available at http://www.microrna.gr/mirgen/.

To accurately characterize the mechanisms of miRNA transcription regulation, Georgakilas et al. constructed DIANA-miRGen v3.0 database to provide accurate TSSs of miRNA genes and the genome-wide maps of TFBSs [30]. According to their previous work [20], they used microTSS algorithm to accurately predict 276 miRNA TSSs. These accurately identified miRNA TSSs and TFBSs are stored in the database. The database DIANA-miRGen v3.0 is available at http://www.microrna.gr/mirgen.

The above databases are all about human and mouse miRNAs. To provide comprehensive information about plant miRNA genes, Chien et al. established the AtmiRNET database [31]. They used high-throughput next-generation sequencing datasets to construct SVM prediction model to predict* Arabidopsis* miRNA TSSs. This database also provides the transcriptional regulation on miRNA genes and putative miRNA-target interactions. In total, 281* Arabidopsis* miRNA TSSs are provided in this study. Among them, 44 miRNAs are intra-miRNA, and this study used TSSs of host genes to define intra-miRNA TSSs. This database is very helpful in that users can understand the transcriptional mechanisms and regulatory functions of miRNA in* A. thaliana*. The AtmiRNET database is freely available at http://AtmiRNET.itps.ncku.edu.tw/.  Table 3 shows the statistics of all five databases discussed above.

## 4. The Analysis of the Construction of the TF-miRNA Regulatory Networks

According to previous studies, most miRNAs are transcribed by noncoding genes, which are also regulated by related transcription factors. It remains unclear how TFs regulate miRNA genes. Constructing the regulatory network of TFs on miRNA genes is a critical step to better understand the functional mechanism of related miRNAs. In recent years, TF-miRNA network has captured increased attentions. People established such network by building computational models or utilizing NGS experiment data.

### 4.1. Computational Methods

Based on Pol II binding patterns around TSSs, Wang et al. developed an approach to predict inter-miRNAs promoter regions [32]. After that, they used position-specific score matrices (PSSM) to predict the TFBSs of STAT1 on genomic regions. Compared with the background promoters nonoverlapped with ChIP-enriched regions of STAT1, it is believed that STAT1 regulates this miRNA if the binding sites are more enriched in specific miRNA promoters. TargetScan was then used for microRNA target prediction to construct the feedback network of STAT1 and miRNAs.

To identify* Arabidopsis* miRNA promoters, Chien et al. established a SVM-based model [33]. First, they paired coexpressed annotated genes with specific miRNAs. By using PWMs from TRANSFAC, they adopted Match program [34] to search TFBSs motifs and defined the coTFBS as the common TFBS motifs that coincided in the promoters of a miRNA and its coexpressed genes. According to the assumption that genes with coexpression pattern may be regulated by the same TFs, the TFs with high frequency of coTFBSs are thought to regulate this miRNA. Finally, the regulatory networks about TFs and miRNAs are visualized by the Cytoscape software.

The previous related studies just provided limited regulatory network of TFs on miRNAs, which restricted the identification of novel TF-miRNA networks. Thus, Falcone et al. developed a software, named infinity, to reveal new regulatory networks of TFs and miRNAs [35]. They collected TSS positions from miRStart and extracted the promoter region sequences of miRNAs from UCSC Genome Browser. This software allows users to search the binding matrix of TFs on the defined promoter regions. This flexibility in this research offers the possibility of establishing unknown TF-miRNAs regulatory networks.

### 4.2. Experimental Evidence-Based Method

Most of the computation methods described above were developed based on the human or mouse genome. Nowadays, people have paid more and more attention to the expressional regulation of other species. Martinez et al. constructed miRNAs regulatory network on the* C. elegans* genome, using high-throughput sequencing technology to experimentally map transcriptional TF-miRNA interactions [36]. For constructing the feedback network of miRNA-TF, they used previous algorithms, such as Pictar [37] and miRanda Targets version 4 [38], to predict the target of miRNAs on specific TFs.

In the meantime, more and more relative databases have been constructed for TF-miRNA regulatory network. For example, TSmiR, constructed by Guo et al., is a database that stores the regulatory networks of TFs and miRNAs in 12 human tissues. Those interactions were derived from the high-throughput experimental data [39]. In total, TSmiR database involves 116 TS miRNAs, 101 TFs, and 2347 TF-miRNA regulatory relations of 12 tissues and is freely available at http://bioeng.swjtu.edu.cn/TSmiR.

TFs and miRNAs are two key elements in the regulation of genes. The regulatory relations, TF-miRNA-target gene, are extremely complex, but they play an important role in pathogenic mechanism of diseases. TFmiR is a web server to collect the coregulatory networks of disease-specific TFs and miRNAs [40]. It integrates genome-wide transcriptional and posttranscriptional regulatory interactions on human diseases, by covering TF-gene, TF-miRNA, miRNA-miRNA, and miRNA gene regulatory networks. In total, TFmiR currently includes the information of almost 10000 genes, 1856 miRNAs, 3000 diseases, and more than 111000 interactions. TFmiR is freely accessible at http://service.bioinformatik.uni-saarland.de/tfmir.

## 5. Discussion

MiRNA has an important role in expressional mechanism of genes, while miRNA also is transcribed by DNA sequences, which is regulated by some special TFs. As we know, promoter regions control the important initiation process of transcription of genes. Accurate identification of the promoter location is significant for better constructing the regulatory networks and understanding the transcriptional mechanisms. Nowadays, plenty of researchers have focused on the prediction of miRNA promoters and have developed many methods. In this review, we summarized these algorithms by two main types, which is either based on the genome sequence features, or based on the high-throughput sequencing technology. The second types based on NGS data used one or mixed features of histone markers, RNA Pol II binding patterns, and nucleosome-free region. The methods based on genome sequence features have limitation in tissues and species which may lead to lower accuracy in different studies. With the development of NGS technology, more and more sequencing datasets will support the models using histone markers, RNA Pol II binding patterns, and nucleosome-free region. They can further improve the prediction accuracy of miRNA promoters.

Plenty of characteristic features that have been used to predict the promoter regions of miRNAs in methods were discussed in this paper, including expressed sequence tags (EST), TSSs, CpG island, TF binding sites, sequence features (N-mer), conservation, histone modification (especially H3K4me3), expression ditags, poly(A) signal, cap analysis of gene expression (CAGE) tags, familial binding profiles (FBP), nucleosome-depleted regions, and GC content (Table 4). We found that a number of models were built based on histone markers which account for the biggest proportion. It indicates that histone markers are key elements for identification of miRNA promoters, especially H3K4me3 enriched in the promoter regions [41].

It is interesting to see that different features can get common prediction for the human miRNA genes at some level after we compared four typical methods shown in Figure 1. But there is no doubt that methods using different features may result in distinct prediction patterns for miRNA promoters. It should be noticed that most of putative results have not had strong experiment evidence to support and verify. In the future, we can exploit more and more NGS data and use machine learning technology to improve the prediction accuracy by selecting appropriate combination of these features.

Benefited from miRNA promoter predictions, regulatory networks of TFs and miRNAs are being constructed. The TF-mediated miRNA regulation network is valuable to better understand the functional mechanisms of most miRNAs. As we discussed in the paper, some models were built based on the computational methods, which can be modified for different tissues, species or diseases, by using appropriate datasets. On the other hand, other models were based on experimental methods. They aimed at one specific tissue, species, or disease according to the experimental design. These models are somehow more accurate in the construction of specific regulatory networks. It is worth integrating the computational method and experimental data to further construct dynamic regulatory networks of TFs and miRNAs.

## Figures and Tables

**Table 1:** Putative miRNA promoter numbers using traditional sequencing data.

Method name	Number of putative human promoters	Number of putative other species promoters					References
EST-extension	41		Rat		Mouse		[13]
			517		162		
CoVote	107	*C. elegans*		*A. thaliana*		*O. sativa*	[14]
		73		95		114	
miPPRs	59	—					[15]

**Table 2:** The 20 common putative miRNA promoters predicted by four models.

Name	Chrom	microRNA position	Marson	X. Wang	Ozsolak	Guohua Wang
hsa-mir-200b	Chr1	1092347–1092441	1088265	1088515	1087712	1088380
hsa-mir-200a	Chr1	1093106–1093195	1088265	1088515	1087791	1088380
hsa-mir-429	Chr1	1094248–1094330	1088265	1088515	1087795	1088380
hsa-mir-92b	Chr1	153431592–153431687	153429179	153429505	153430515	153430271
hsa-mir-148a	Chr7	25956064–25956131	25955148	25957430	25957069	25957227
hsa-mir-182	Chr7	129197459–129197568	129204548	129206490	129206638	129207158
hsa-mir-96	Chr7	129201768–129201845	129204548	129206490	129206331	129207158
hsa-mir-183	Chr7	129201981–129202090	129204548	129206490	129206299	129207158
hsa-let-7a-1	Chr9	95978060–95978139	95968291	95968360	95967305	95968990
hsa-let-7f-1	Chr9	95978450–95978536	95968291	95968360	95967585	95968990
hsa-let-7d	Chr9	95980937–95981023	95968291	95968360	95967585	95968990
hsa-mir-345	Chr14	99843949–99844046	99840674	99842750	99840834	99843433
hsa-mir-484	Chr16	15644652–15644730	15643092	15644680	15643760	15644503
hsa-mir-99b	Chr19	56887677–56887746	56883717	56884440	56882679	56884486
hsa-let-7e	Chr19	56887851–56887929	56883717	56884440	56884876	56884486
hsa-mir-125a	Chr19	56888319–56888404	56883717	56884440	56883012	56884486
hsa-mir-659	Chr22	36573631–36573727	36573792	36575350	36574528	36575388
hsa-mir-545	ChrX	73423664–73423769	73426274	73428915	73428342	73428923
hsa-mir-374a	ChrX	73423846–73423917	73426274	73428915	73428487	73428923
hsa-mir-505	ChrX	138833973–138834056	138840014	138842900	138842217	138842643

**Table 3:** The number of miRNA promoters in five databases.

Database	miRNAs	Inter-miRNAs	Intra-miRNAs	Species
miRStart	940	352	588	Human
miRT	588	206	382	Human
DIANA-miRGen	428	428	0	Human, mouse
miRGen	1189	766	423	Human, mouse
AtmiRNET	281	237	44	Arabidopsis

**Table 4:** The features used in the miRNA promoter prediction models.

Literature	EST	N-mer	TATA box	CAAT box	GC box	CpG island	Conservation	TFBS	DNase I	Histone marker	Pol II	Nucleosome
[13]	√											
[14]		√										
[15]			√	√	√							
[16]	√		√			√	√					
[17]		√				√						
[9]										√		
[8]		√						√		√		
[18]										√		
[10]											√	
[11]											√	
[19]											√	
[20]									√	√	√	
[7]						√	√	√		√		√
